# Association of Prediagnostic Frailty, Change in Frailty Status, and Mortality After Cancer Diagnosis in the Women’s Health Initiative

**DOI:** 10.1001/jamanetworkopen.2020.16747

**Published:** 2020-09-14

**Authors:** Elizabeth M. Cespedes Feliciano, Chancellor Hohensee, Ashley E. Rosko, Garnet L. Anderson, Electra D. Paskett, Oleg Zaslavsky, Robert B. Wallace, Bette J. Caan

**Affiliations:** 1 Division of Research, Kaiser Permanente Northern California, Oakland; 2 Division of Public Health Sciences, Fred Hutchinson Cancer Research Center, Seattle, Washington; 3 Division of Hematology, Department of Internal Medicine, The Ohio State University, Columbus; 4 Division of Cancer Prevention and Control, Department of Internal Medicine, The Ohio State University, Columbus; 5 School of Nursing, University of Washington, Seattle; 6 Department of Epidemiology, College of Public Health, The University of Iowa, Iowa City

## Abstract

**Importance:**

Understanding changes in frailty in relation to cancer diagnosis can inform optimal selection of cancer treatments and survivorship care.

**Objective:**

To investigate associations of prediagnostic frailty and change in frailty status with mortality after a cancer diagnosis.

**Design, Setting, and Participants:**

This multicenter, prospective cohort study included 7257 community-dwelling, postmenopausal women in the United States who had frailty assessed at the Women’s Health Initiative (WHI) enrollment (1993-1998) and the 3-year visit who were subsequently diagnosed as having invasive cancer. The data were analyzed from January 7, 2019, to June, 8, 2020.

**Exposure:**

Frailty scores were defined from validated questionnaire items conceptually aligned with the Fried frailty phenotype, including at least 3 of the following characteristics: self-reported unintentional weight loss, exhaustion, low physical activity, and muscle weakness or impaired walking. Physical function components of the frailty score were updated a median of 10 (range, 1-18) times.

**Main Outcomes and Measures:**

Using multivariable-adjusted Cox proportional hazards models, this study examined associations of prediagnostic frailty (at the 3-year visit, before cancer diagnosis) and prediagnostic changes in frailty (from enrollment to the 3-year visit) with mortality. Women were followed up beginning from cancer diagnosis for mortality outcomes through March 2018. In linear mixed-effects models with frailty scores as a function of time since cancer diagnosis, this study evaluated whether the time slope, ie, the rate of change in frailty score, increased after cancer diagnosis.

**Results:**

This study included 7257 women in the WHI cohort who completed frailty assessments at enrollment and the 3-year WHI visit before cancer diagnosis and subsequently developed cancer. Cancer cases included 2644 breast cancers (36%), 822 lung cancers (11%), 691 colorectal cancers (10%), 445 endometrial cancers (6%), and 286 ovarian cancers (4%). At the 3-year visit, prior to cancer diagnosis, the mean (SD) age was 63 (7) years, and 1161 of 7257 (16%) of participating women met criteria for frailty; 2129 of 7257 (29%) were prefrail, and 3967 of 7257 (55%) were nonfrail. Over a median follow-up of 5.8 years after cancer diagnosis (range, 1 day to 19.9 years), 3056 women died. After multivariable adjustment, women who were frail (vs nonfrail) before cancer diagnosis had an increased risk of mortality after cancer diagnosis (hazard ratio [HR], 1.40; 95% CI, 1.26-1.55; *P* for trend <.001). Sustained frailty (21% [1537 of 7257] of women) or worsening frailty (22% [1578 of 7257]) vs being consistently nonfrail (45% [3266 of 7257]) before cancer diagnosis increased the risk of mortality after cancer diagnosis (HR, 1.25; 95% CI, 1.14-1.38 and 1.22; 95% CI, 1.11-1.34, respectively; *P* for trend <.001). In linear mixed-effects models, the rate of increase in physical frailty over time was statistically significantly higher after cancer diagnosis.

**Conclusions and Relevance:**

Sustained and worsening frailty before cancer diagnosis was associated with an increased risk of mortality after cancer diagnosis in postmenopausal women. Furthermore, the rate of decline in physical function accelerated after cancer diagnosis. Frailty assessment could provide valuable information and perhaps prompt interventions to reduce and preempt worsening of physical frailty after cancer diagnosis.

## Key Points

**Question:**

Are prediagnostic physical frailty and change in frailty status over time associated with mortality after a cancer diagnosis?

**Findings:**

In this cohort study of 7257 postmenopausal women with cancer, sustained and worsening frailty before a cancer diagnosis was associated with a more than 20% increased risk of mortality after cancer diagnosis. Furthermore, the rate of decline in physical function accelerated after cancer diagnosis.

**Meaning:**

These data suggest that frailty assessment in patients with cancer could provide valuable prognostic information and perhaps prompt interventions to reduce or preempt worsening of physical frailty after a cancer diagnosis.

## Teaser

This cohort study investigates associations of prediagnostic frailty and change in frailty status with mortality after cancer diagnosis.

## Introduction

There are 16 million cancer survivors in the United States, and 60% are 65 years or older; by 2040, the number of survivors will increase to 26 million, with 73% being 65 years and older.^1^ Given this demographic shift, interest is growing in how age and age-related comorbidities alter cancer survival and how cancer and its treatments change trajectories of healthy aging. Frailty, diminished physiologic reserve that results in decreased resiliency and adaptive capacity and increased vulnerability to stressors,^2^ is an aging syndrome prevalent among older adults that is associated with morbidity and mortality.^3,4^

Two widely accepted frailty paradigms are the frailty phenotype and the cumulative deficit model. Although these assessments overlap to some degree, each has advantages. The frailty phenotype uses the Fried criteria to describe a clinically reproducible syndrome that includes weight loss, exhaustion, low physical activity, slowness, and weakness.^5,6,7^ The Fried frailty phenotype can be evaluated at first contact to stratify patients as nonfrail, prefrail, or frail. The cumulative deficit model is assessed by continuous scores (eg, the Rockwood index) that tally accumulated physical and cognitive deficits arising from diseases and disabilities.^8,9,10,11^ Such indexes provide granular assessment that is practical in retrospective medical record cohorts.

Although the prevalence of frailty varies by the definition used, more than one-half of older patients with cancer are frail or prefrail at cancer diagnosis; these patients are thought to be at increased risk of chemotherapy intolerance, postoperative complications, and mortality.^12^ In addition to preexisting frailty, cancer and its treatments present additional stressors that can accelerate aging and worsen frailty status.^5,7,13,14,15^ Understanding the association of frailty and change in frailty status with cancer diagnosis and subsequent mortality is important for optimal selection of cancer treatments and survivorship care.^3,4^ Despite the dynamic nature of frailty, to our knowledge, no prior study has examined whether changes in functional decline over time are associated with cancer mortality.

To fill this gap, the present study included 7257 participants in the Women’s Health Initiative (WHI) who developed invasive cancer. At enrollment and 3 years after enrollment, frailty scores were defined from validated questionnaire items conceptually aligned with the Fried frailty phenotype, including at least 3 of the following characteristics: self-reported unintentional weight loss, exhaustion, low physical activity, and muscle weakness or impaired walking. Women exhibiting up to 2 of these characteristics were considered prefrail.^5,6,7^ We then examined associations of prediagnostic frailty (at the 3-year visit, before cancer diagnosis) and prediagnostic changes in frailty (from enrollment to the 3-year visit) with mortality. Women were followed up beginning from cancer diagnosis for mortality outcomes through March 2018. Secondarily, we examined whether the rate of increase in frailty changed after cancer diagnosis.

## Methods

### Study Population

The design and conduct of the WHI has been previously described.^16,17^ In brief, the WHI observational study (N = 93 676) included postmenopausal women aged 50 to 79 years from 40 US clinical centers between 1993 and 1998. At enrollment, self-administered questionnaires were used to collect information on demographic characteristics; medical, reproductive, and family history; and lifestyle factors, including smoking and physical activity. Written informed consent materials were reviewed by study investigators, local institutional review boards, the WHI data and safety monitoring board, the WHI policy advisory committee, the National Institutes of Health, the Institute of Medicine, focus groups of postmenopausal women, and media.^18^ Written informed consent was obtained from all participants, and this study was approved by each institution’s institutional review board. This study followed the Strengthening the Reporting of Observational Studies in Epidemiology (STROBE) reporting guideline.

For this prospective cohort study, our main exposure was defined as frailty assessed at the 3-year WHI visit (so as to examine change in frailty). Eligible participants were enrolled in the WHI (N = 93 676) and had a first diagnosis of cancer after the 3-year visit (n = 11 913). Women were excluded from this set of 11 913 if they reported a history of cancer before WHI enrollment (1993-1998), were missing covariate data, or were missing frailty status at enrollment or at the 3-year WHI visit (n = 4656). Therefore, after exclusions, this multicenter study included 7257 US community-dwelling, postmenopausal women with frailty assessed at 2 time points before diagnosis of invasive cancer. Women included vs women excluded were similar with respect to age, race/ethnicity, and body mass index (calculated as weight in kilograms divided by height in meters squared). Data analysis was conducted from January 7, 2019, to June, 8, 2020.

### Exposures

The main exposure was a frailty score, which was defined as in prior publications^5,6,7^ using validated questionnaire items to operationalize components of the Fried frailty phenotype. At enrollment, these components included self-reported unintentional weight loss of at least 15 lb (6.8 kg [to convert lbs to kg, multiply by 0.45]) in the past 6 months, self-reported low physical activity (<2.5 metabolic equivalent task hours per week), and muscle weakness or impaired walking and exhaustion (measured using the 36-Item Short Form Health Survey [SF-36] physical function [<70] and vitality [<55] scores).^19,20,21^ These measures were repeated at the 3-year WHI visit, with unintentional weight loss redefined based on measured weight loss at the clinic visit exceeding 5% since enrollment and no self-reported indication of an intention to lose at least 5 lb in the past 2 years. As in prior publications,^5,6,7^ for each measure (except unintentional weight loss), a frailty component was classified as present if the participant had a score in the lowest quartile of the distribution for that component. To align the scoring with the Fried criteria, poor physical function was scored as 2 points because the SF-36 measures both muscle strength and walking ability components. The number of frailty components present was summed, yielding a range of 0 to 5. A frailty cut point of at least 3 was used, with women scoring 1 to 2 considered prefrail and those scoring 0 considered robust or nonfrail.^5,6,7^ Change in frailty was computed as a continuous change in score from enrollment to the 3-year WHI visit and as a categorical variable indicating an increase or a decrease or that the woman scored as frail or nonfrail both times.

The eFigure in the Supplement shows the time line for frailty data collection in the WHI and the analytic time line for our analysis. All women had frailty scores measured at 2 time points before cancer diagnosis (WHI enrollment and the 3-year visit). In addition, most women participated in the WHI extension studies (6318 [87%] in 2005-2010 and 5157 [71%] in 2010-2015) and thus had updated measurements available for time-varying analyses. The physical function components of the frailty score assessed using the SF-36 were updated annually, whereas the energy and fatigue components were updated only once, in 2012. Weight change and physical activity were updated only at enrollment and the 3-year visit. Overall, physical function components of the frailty score were updated a median of 10 (range, 1-18) times; among women participating in the extension studies, the median number of frailty score updates was 12 (range, 1-18).

### Outcomes

Annually, newly reported cancer diagnoses were documented with medical records and confirmed and coded at the WHI Clinical Coordinating Center.^22^ Deaths were ascertained through numerous sources (eg, reports from relatives, returned mail, and obituary notices) and documented with medical records or death certificates. Serial National Death Index queries through March 2018 provided death certificate information regardless of reconsent status, resulting in overall survival information that is 98% complete.^23^

### Covariates

Using a standardized approach, trained research staff measured participants’ height and weight, from which we computed body mass index. Via questionnaires, participants reported total physical activity, pack-years of smoking, educational attainment, family income, diagnosis of or treatment for diabetes, and any family history of cancer. In addition, we calculated the Charlson Comorbidity Index score for each participant at the 3-year visit and the prevalence of individual chronic conditions associated with frailty, including history of rheumatoid arthritis, history of congestive heart failure, emphysema, chronic obstructive pulmonary disease (COPD), liver disease, and chronic kidney disease.

### Statistical Analysis

All analyses were conducted using R 3.4.4 (R Project for Statistical Computing), with 2-sided α = .05 as the threshold for statistical significance. Using multivariable-adjusted Cox proportional hazards models, this study calculated the hazard ratios (HRs) for mortality associated with prediagnostic frailty (at the 3-year WHI visit, before cancer diagnosis) and prediagnostic changes in frailty (from enrollment to the 3-year WHI visit). Analyses included women with cancer followed up from the time of diagnosis (time zero, as shown in the eFigure in the Supplement) to death or censoring (loss to follow-up or March 2018). Models for mortality after cancer diagnosis were stratified by age at enrollment (first exposure assessment) and adjusted for the covariates above, as well as for continuous age, cancer stage at diagnosis (local, regional, or distant), and year of diagnosis. When examining specific cancer sites, we adjusted for prognostic indicators specific to that cancer site. These included hormone receptor and ERBB2 (formerly HER2) status for breast cancer, non–small cell vs small cell for lung cancer, serous vs nonserous for ovarian cancer, colon vs rectum for colorectal cancer, and type 1 endometrioid adenocarcinomas vs type 2 uterine serous and clear cell carcinomas for endometrial cancer. We repeated these analyses with 3-year prediagnostic change in frailty status from enrollment to the 3-year WHI visit as the main exposure. As stated previously, all analyses excluded women who developed cancer before the 3-year WHI visit.

In exploratory analyses examining whether women who develop cancer become increasingly frail, we fit linear mixed-effects models with frailty scores as a function of time since cancer diagnosis to calculate the time slope of change in the frailty score from enrollment through all subsequent measurements available as part of the WHI extension studies. These models included a random intercept and random slope for the change in frailty score as a function of time since diagnosis. We also included a time-varying indicator for date of first cancer diagnosis, at which time slopes could change. We then tested whether the rate of change in frailty (time slope and 95% CI) was altered from before to after cancer diagnosis using a likelihood ratio test to examine whether the addition of a linear spline with a knot at date of cancer diagnosis fit the data better than a more parsimonious model without a slope change. Subsequently, we evaluated whether the change in slope remained statistically significant after controlling for covariates (race/ethnicity, body mass index, pack-years of smoking, educational attainment, Charlson Comorbidity Index score, and any family history of cancer) and age and cancer stage at diagnosis. The physical function component of the SF-36 was most frequently updated; therefore, longitudinal assessments can be interpreted as the rate of change per year in physical function from before to after cancer diagnosis.

For some women, frailty metrics at the 3-year WHI visit were far from cancer diagnosis. The median time from the 3-year WHI visit to cancer diagnosis was 7.4 years (range, 1 day to 19.8 years). To address this gap, we fit Cox proportional hazards models with time-updated frailty scores as the exposure using all available measurements to assess whether frailty closer to diagnosis was more strongly associated with survival (the median time from diagnosis to the closest prediagnostic frailty assessment was 1.6 years [range, 1 day to 17.3 years]). Finally, we considered whether our results were consistent across strata defined by age and cancer stage at diagnosis (local, regional, or distant) or excluding participants with comorbid conditions associated with frailty that were prevalent at the 3-year WHI visit (history of rheumatoid arthritis, history of congestive heart failure, emphysema, COPD, liver disease, and chronic kidney disease).

## Results

This study included 7257 women in the WHI cohort who completed frailty assessments at enrollment and the 3-year WHI visit before cancer diagnosis and subsequently developed cancer. Cancer cases included 2644 breast cancers (36%), 822 lung cancers (11%), 691 colorectal cancers (10%), 445 endometrial cancers (6%), and 286 ovarian cancers (4%). At the time of the 3-year WHI visit (prediagnostic) frailty assessment, the mean (SD) age was 63 (7) years, and 44% (3205 of 7257) of women were 65 years or older. Sixteen percent (1161 of 7257) of participating women with cancer met the criteria for frailty, 29% (2129 of 7257) were prefrail, and 55% (3967 of 7257) were nonfrail. Women who were frail (vs nonfrail) at the 3-year WHI visit were older, more likely to be obese, and less likely to have a college education or a family income of $75 000 or more per year (eTable in the Supplement).

**Figure 1.**
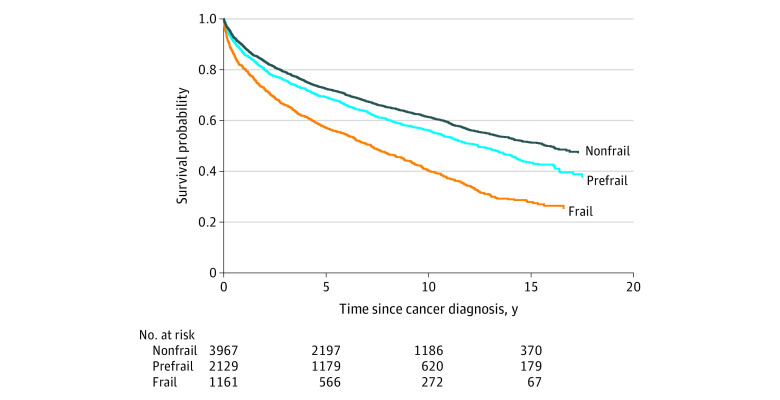
Overall Survival Probability After Cancer Diagnosis Among 7257 Women in the WHI Study by Prediagnostic Frailty Status WHI indicates Women’s Health Initiative.

Over a median follow-up 5.8 years (range, 1 day to 19.9 years) from cancer diagnosis, 3056 women (42%) died. Most deaths were from cancer (1985 [65%]), although the second most common cause of death was cardiovascular disease (314 [10%]). The survival probability after cancer diagnosis was lowest among frail women, intermediate among prefrail women, and highest among women who were nonfrail at enrollment (Figure 1). After multivariable adjustment, women who were frail (vs nonfrail) before cancer diagnosis had an increased risk of mortality after cancer diagnosis (HR, 1.40; 95% CI, 1.26-1.55; *P* for trend <.001) (Table 1).

**Table 1.** Association of Prediagnostic Frailty With Mortality After Cancer Diagnosis Among 7257 Women in the WHI Study^a^

Exposure	HR (95% CI)	*P* for trend	No. of events (n = 3056)	No. of women (N = 7257)	Rate per person-years of follow-up
All cancers					
Frail	1.40 (1.26-1.55)	<.001	687	1161	0.10
Prefrail	1.12 (1.03-1.22)		920	2129	0.06
Nonfrail	1 [Reference]		1449	3967	0.05
Breast					
Frail	1.74 (1.39-2.16)	<.001	156	391	0.05
Prefrail	1.32 (1.09-1.60)		201	774	0.03
Nonfrail	1 [Reference]		272	1479	0.02
Lung					
Frail	1.33 (1.05-1.68)	.02	144	178	0.29
Prefrail	1.09 (0.89-1.34)		170	239	0.20
Nonfrail	1 [Reference]		264	405	0.18
Colorectal					
Frail	1.93 (1.39-2.69)	<.001	78	126	0.10
Prefrail	1.32 (0.99-1.76)		87	213	0.06
Nonfrail	1 [Reference]		132	352	0.05
Ovarian					
Frail	1.34 (0.77-2.32)	.44	30	37	0.25
Prefrail	1.00 (0.67-1.51)		43	72	0.11
Nonfrail	1 [Reference]		103	177	0.12
Endometrial					
Frail	2.08 (1.17-3.71)	.03	31	55	0.07
Prefrail	1.19 (0.76-1.85)		41	145	0.03
Nonfrail	1 [Reference]		51	245	0.02

Abbreviations: HR, hazard ratio; WHI, Women’s Health Initiative.

^a^ Mortality included all confirmed deaths after a cancer diagnosis that occurred after the 3-year WHI visit and were reported and confirmed through March 2018. All Cox proportional hazards models adjusted for the following covariates at the 3-year WHI visit: race/ethnicity, body mass index, pack-years of smoking, educational attainment, Charlson Comorbidity Index score, any family history of cancer, and continuous age and stage at cancer diagnosis. *P* for trend was calculated for frailty categories as an ordinal variable.

**Figure 2.**
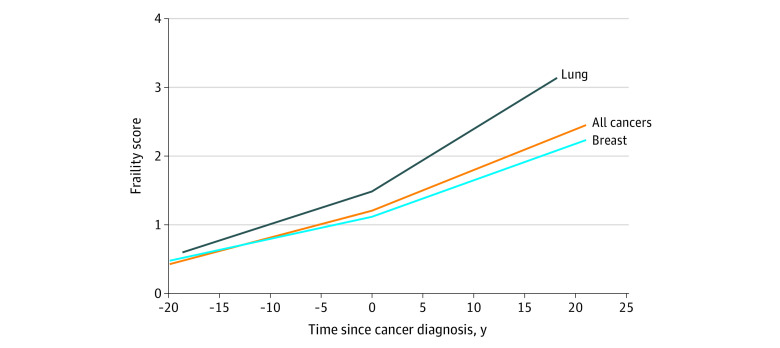
Rate of Increase in Physical Frailty From Before to After Cancer Diagnosis Among 7257 Women in the WHI Study Physical frailty assessments are based on the frailty score at the 3-year Women’s Health Initiative (WHI) study visit and subsequent updates to the physical function component of the frailty score. Analyses are restricted to women diagnosed as having cancer. Shown is the change in frailty score in relation to cancer diagnosis from a linear mixed-effects model with a piecewise linear spline (knot at date of cancer diagnosis) and a random intercept and slope for each individual.

Over the 3 years after WHI enrollment, 21% (1537 of 7257) of women had sustained frailty (categorized as frail at both times), 22% (1578 of 7257) of women had worsening frailty (eg, from nonfrail to prefrail or frail and from prefrail to frail), and 12% (876 of 7257) had improved frailty (eg, from frail to prefrail or nonfrail and from prefrail to nonfrail). Women with sustained frailty or worsening frailty vs those who were consistently nonfrail (45% [3266 of 7257]) before cancer diagnosis had an increased risk of mortality after cancer diagnosis (HR, 1.25; 95% CI, 1.14-1.38 and 1.22; 95% CI, 1.11-1.34, respectively; *P* for trend <.001) (Table 2). Furthermore, cancer diagnosis served as an inflection point, after which women became increasingly frail. The rate of increase in physical frailty over time was statistically significantly higher after cancer diagnosis (Figure 2).

**Table 2.** Association of Prediagnostic Change in Frailty With Mortality After Cancer Diagnosis Among 7257 Women in the WHI Study^a^

Exposure	HR (95% CI)	*P* for trend	No. of events (n = 3056)	No. of women (N = 7257)	Rate per person-years of follow-up
All cancers					
Decrease in frailty score	1.08 (0.95-1.22)	<.001	357	876	0.06
Frail at both times	1.25 (1.14-1.38)		735	1537	0.07
Increase in frailty score	1.22 (1.11-1.34)		784	1578	0.08
Nonfrail at both times	1 [Reference]		1180	3266	0.05
Continuous change	1.07 (1.03-1.10)	<.001	3056	7257	
Breast					
Decrease in frailty score	1.07 (0.80-1.43)	.01	64	303	0.03
Frail at both times	1.46 (1.18-1.81)		168	565	0.04
Increase in frailty score	1.41 (1.15-1.74)		171	543	0.04
Nonfrail at both times	1 [Reference]		226	1233	0.02
Continuous change	1.10 (1.02-1.18)	.01	629	2644	
Lung					
Decrease in frailty score	1.01 (0.77-1.32)	.26	84	114	0.20
Frail at both times	1.24 (0.98-1.58)		133	175	0.23
Increase in frailty score	1.19 (0.95-1.49)		160	211	0.25
Nonfrail at both times	1 [Reference]		201	322	0.17
Continuous change	1.07 (1.00-1.14)	.05	578	822	
Colorectal					
Decrease in frailty score	0.86 (0.55-1.33)	.10	28	82	0.05
Frail at both times	1.69 (1.22-2.34)		79	157	0.08
Increase in frailty score	1.47 (1.08-2.01)		78	162	0.07
Nonfrail at both times	1 [Reference]		112	290	0.06
Continuous change	1.14 (1.03-1.28)	.02	297	691	
Ovarian					
Decrease in frailty score	1.29 (0.76-2.19)	.23	20	36	0.13
Frail at both times	1.07 (0.64-1.80)		32	48	0.15
Increase in frailty score	1.27 (0.82-1.98)		37	56	0.14
Nonfrail at both times	1 [Reference]		87	146	0.12
Continuous change	1.10 (0.94-1.29)	.25	176	286	
Endometrial					
Decrease in frailty score	0.94 (0.47-1.86)	.10	14	59	0.03
Frail at both times	1.06 (0.62-1.81)		32	93	0.04
Increase in frailty score	1.61 (0.98-2.64)		36	95	0.05
Nonfrail at both times	1 [Reference]		41	198	0.02
Continuous change	1.27 (1.04-1.55)	.02	123	445	

Abbreviations: HR, hazard ratio; WHI, Women’s Health Initiative.

^a^ Mortality included all confirmed deaths after a cancer diagnosis that occurred after the 3-year WHI visit and were reported and confirmed through March 2018. All Cox proportional hazards models adjusted for the following covariates at the 3-year WHI visit: race/ethnicity, body mass index, pack-years of smoking, educational attainment, Charlson Comorbidity Index score, any family history of cancer, and continuous age and stage at cancer diagnosis. *P* for trend was calculated for frailty categories as an ordinal variable.

When examining cancers separately, there was little evidence that the patterns of association differed by cancer site. Once diagnosed, frail women had a higher risk of mortality than nonfrail women for every cancer site. Similar results were observed for change in frailty status (Table 2).

**Figure 3.**
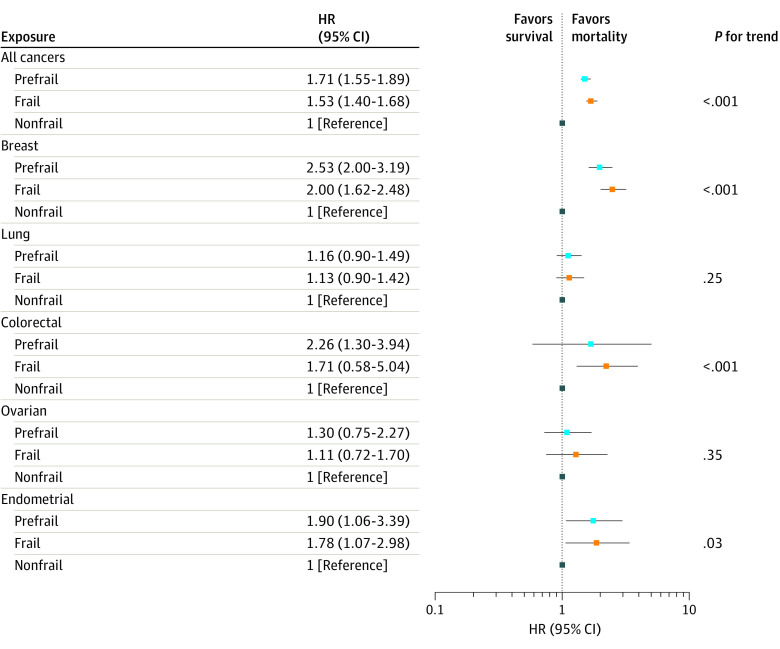
Time-Varying Frailty Status and Mortality After Cancer Diagnosis Among 7257 Women in the Women’s Health Initiative (WHI) Study Frailty scores are a time-varying exposure, with nonfrail as the reference. All Cox proportional hazards models adjusted for the following covariates at the 3-year WHI visit: race/ethnicity, body mass index, pack-years of smoking, educational attainment, Charlson Comorbidity Index score, and any family history of cancer. *P* for trend was calculated for frailty categories as an ordinal variable. All analyses were restricted to women without a prior cancer diagnosis at the 3-year WHI visit who were followed up for mortality beginning from the date of their first cancer diagnosis through March 2018. HR indicates hazard ratio.

When we used time-updated assessments reflective of physical function closer to cancer diagnosis (Figure 3), results were consistent, and, in some cases, the risk of mortality associated with frailty was even greater. For example, in time-updated models, frail (vs nonfrail) women had a 71% increased risk of mortality after cancer diagnosis (HR, 1.71; 95% CI, 1.55-1.89).

Frailty was consistently associated with an increased risk of mortality across subgroups defined by age at cancer diagnosis. The HRs of mortality for frail (vs nonfrail) women were 1.66 (95% CI, 1.16-2.38) for women younger than 65 years (n = 876), 1.43 (95% CI, 1.21-1.70) for women aged 65 to 74 years (n = 3013), and 1.37 (95% CI, 1.20-1.56) for women 75 years or older (n = 3368). Analyses stratified by cancer stage at diagnosis also yielded similar associations for local (n = 3856), regional (n = 1540), and distant (n = 1861) cancer stage at diagnosis. Restricting analyses to participants without history of rheumatoid arthritis, history of congestive heart failure, emphysema, COPD, liver disease, or chronic kidney disease at the 3-year WHI visit (n = 3445) did not materially change our results.

## Discussion

In this study of 7257 postmenopausal women who developed cancer, prediagnostic frailty was associated with a 40% (HR, 1.40; 95% CI, 1.26-1.55) increased risk of mortality after cancer diagnosis (after adjusting for potential confounders). Similarly, sustained frailty (HR, 1.25; 95% CI, 1.14-1.38) or worsening frailty (HR, 1.22; 95% CI, 1.11-1.34) before cancer diagnosis was associated with a more than 20% increased risk of mortality after cancer diagnosis. Cancer diagnosis served as an inflection point, after which the rate of increase in frailty over time accelerated, reflecting a decline in physical function. Our study reinforces the importance of assessing and addressing frailty in patients with cancer.

Prior studies^3,12^ have found that frailty is highly prevalent among patients with cancer and, as in the general population, is associated with increased treatment complications and postoperative and 1-year or 5-year mortality. Although heterogeneity in frailty definitions and outcomes makes direct comparison with prior studies challenging, our findings support those earlier results by documenting an increased long-term risk of mortality among patients with cancer who are frail and, for the first time to our knowledge, examining change in frailty status. Previous studies in patients with cancer have used other operational definitions of frailty, such as the cumulative deficit model^24^ or comprehensive geriatric assessments,^25^ both of which consider a broader range of functional ability and physical and cognitive health. We chose to evaluate a score conceptually aligned with the Fried criteria for frailty, which predominantly assesses physical frailty (our interest in this study) and has been shown to be sensitive to change after interventions in frail patients.^26^ Furthermore, the Fried frailty phenotype has been widely adopted and adapted because of its good construct validity^6^ and agreement with other approaches for assessing frailty.^27^

To our knowledge, this study is the first to report that worsening prediagnostic frailty is detrimental to cancer survival. In addition, we found that the rate of increase in frailty (reflecting functional decline) accelerated after cancer diagnosis. Cancer and its treatments are hypothesized to accelerate aging, associated with increases in inflammation, cellular senescence, and other biological hallmarks of aging that underlie physical frailty.^5,7,13,14,15^ We found support for this hypothesis in that the rate of increase in functional decline over time accelerated after cancer diagnosis. This finding is consistent with prior literature demonstrating that functional status and overall health decline steeply after cancer diagnosis compared with age-matched controls without cancer.^28,29^ Although examining cancer treatments was outside the scope of the present analysis, much of this accelerated aging has been hypothesized to be associated with chemotherapy and radiotherapy, which shorten telomere length and increase p16 expression, proinflammatory cytokines, and resting energy expenditure.^30^

In addition to screening for frailty in oncology settings, exercise and physical activity interventions may be beneficial in this setting. Frailty is modifiable, as evidenced by the fact that in this study a substantial proportion of women changed frailty categories over a 3-year period. In the general population, physical activity interventions (eg, resistance and aerobic exercise, brisk walking, or balance training) help to mitigate physical frailty and maintain independence in older adults.^31^ Nutrition support may also play a role, although results of multimodal interventions combining nutrition and physical activity have been mixed.^32^ Among cancer survivors, physical activity interventions are effective in preserving or increasing physical function,^33^ and findings of observational studies^34,35,36,37,38,39^ suggest that exercise may reduce mortality. Such supportive interventions might be considered if a patient is screened as frail at cancer diagnosis.

### Strengths and Limitations

This study has notable strengths. It is one of the largest studies to date to examine phenotypic frailty and long-term cancer mortality. In addition, to our knowledge, it is one of the only studies to examine changes in functional decline over time.

This study also has some limitations. First, frailty assessments were not timed to correspond to cancer diagnosis; therefore, the median time from the 3-year WHI visit frailty assessment to cancer diagnosis was 7.4 years. However, in analyses leveraging repeated frailty assessments collected over subsequent decades, the results were not only consistent but also indicated a stronger increased risk of mortality associated with prediagnostic frailty; this finding suggests that, while even measurements of frailty collected long before cancer diagnosis contain useful information, the closer in time to cancer diagnosis that frailty is assessed the stronger its association with cancer outcomes. Second, residual confounding is a possibility in all observational studies. Although we examined associations separately by cancer site and adjusted for age and stage at cancer diagnosis, preclinical cancer (ie, undiagnosed cancer that has not yet been detected clinically) could have contributed to frailty onset (eg, via unintentional weight loss). Although older age is associated with both frailty and mortality risk, we controlled for age at frailty assessment and age at cancer diagnosis in analyses and found similar associations across subgroups defined by age at cancer diagnosis. Data collection on specific cancer treatment modalities is ongoing. Further investigation on whether the observed accelerated functional decline after cancer diagnosis is because of cancer or its treatments is a critical area for future research.

## Conclusions

This study found that sustained and worsening frailty before cancer diagnosis was associated with an increased risk of mortality after cancer diagnosis. Furthermore, the rate of decline in physical function accelerated after cancer diagnosis. These data support the importance of frailty assessment in patients with cancer to reduce and preempt worsening of physical frailty after cancer diagnosis.

## Notes

Supplement. **eTable.** Participant Characteristics by Frailty Status in the Women’s Health Initiative (n = 7,257) **eFigure.** Timelines for Frailty Data Collection Beginning at Women’s Health Initiative Enrollment Onwards and for Analysis of Time to Death Following Cancer Diagnosis
