# Supplementary material for: Association of Prediagnostic Frailty, Change in Frailty Status, and Mortality After Cancer Diagnosis in the Women’s Health Initiative
Source: JAMA Netw Open. 2020 Sep 14;3(9):e2016747. doi: 10.1001/jamanetworkopen.2020.16747 (PMC7490646; doi:10.1001/jamanetworkopen.2020.16747)
Supplement: Supplement. — eTable. Participant Characteristics by Frailty Status in the Women’s Health Initiative (n = 7,257) eFigure. Timelines for Frailty Data Collection Beginning at Women’s Health Initiative Enrollment Onwards and for Analysis of Time to Death Following Cancer Diagnosis [file jamanetwopen-e2016747-s001.pdf]

Supplementary Online Content

Cespedes Feliciano EM, Hohensee C, Rosko AE, et al. Association of prediagnostic frailty, change in frailty status, and mortality after cancer diagnosis in the Women's Health Initiative. *JAMA Netw Open*. 2020;3(9):e2016747. doi:10.1001/jamanetworkopen.2020.16747

eTable. Participant Characteristics by Frailty Status in the Women's Health Initiative (n=7,257)

eFigure. Timelines for Frailty Data Collection Beginning at Women's Health Initiative Enrollment Onwards and for Analysis of Time to Death Following Cancer Diagnosis

This supplementary material has been provided by the authors to give readers additional information about their work.

© 2020 Cespedes Feliciano EM et al. *JAMA Network Open*.

eTable. Participant Characteristics by Frailty Status in the Women's Health Initiative (n=7,257) [a]

	Overall	Non-frail	Pre-Frail	Frail
N	7257	3967	2129	1161
Frailty score at baseline, mean (sd)	0.94 (1.28)	0.00 (0.00)	1.32 (0.47)	3.45 (0.55)
Baseline physical functioning, mean (sd)[b]	83.80 (18.08)	91.00 (10.65)	82.81 (16.06)	61.06 (22.08)
Baseline energy/fatigue, mean (sd)[b]	65.51 (18.66)	72.83 (13.97)	60.84 (18.52)	49.05 (19.67)
Total physical activity, MET-hours, mean	14.30 (13.99)	18.24 (14.69)	10.71 (12.07)	7.42 (9.72)
3-yr change in weight (kg), mean (sd)	0.01 (0.12)	0.02 (0.12)	0.01 (0.11)	0.01 (0.12)
Age group (5 yr intervals), %				
50-54	870 (12.0)	479 (12.1)	291 (13.7)	100 (8.6)
55-59	1435 (19.8)	872 (22.0)	401 (18.8)	162 (14.0)
60-64	1747 (24.1)	988 (24.9)	505 (23.7)	254 (21.9)
65-69	1680 (23.2)	927 (23.4)	457 (21.5)	296 (25.5)
70-74	1097 (15.1)	534 (13.5)	342 (16.1)	221 (19.0)
75-79	428 (5.9)	167 (4.2)	133 (6.2)	128 (11.0)
Race/ethnicity, %				
American Indian/ Alaska Native	29 (0.4)	11 (0.3)	6 (0.3)	12 (1.0)
Asian/pacific Islander	142 (2.0)	83 (2.1)	41 (1.9)	18 (1.6)
Hispanic/Latina	121 (1.7)	50 (1.3)	41 (1.9)	30 (2.6)
Non-Hispanic Black	289 (4.0)	132 (3.3)	88 (4.1)	69 (5.9)
Non-Hispanic White	6592 (90.8)	3654 (92.1)	1923 (90.3)	1015 (87.4)
Unknown	84 (1.2)	37 (0.9)	30 (1.4)	17 (1.5)
Body-mass index categories (kg/m^2), %				
<25	3123 (43.0)	2054 (51.8)	803 (37.7)	266 (22.9)
25 - <30	1665 (22.9)	572 (14.4)	569 (26.7)	524 (45.1)
>=30	2469 (34.0)	1341 (33.8)	757 (35.6)	371 (32.0)
Waist circumference (cm), mean (sd)	84.40 (13.29)	81.22 (11.29)	85.64 (13.33)	92.95 (15.24)
Smoking status, %				
Current	529 (7.3)	214 (5.4)	192 (9.0)	123 (10.6)
Never	3461 (47.7)	1911 (48.2)	1025 (48.1)	525 (45.2)
Past	3267 (45.0)	1842 (46.4)	912 (42.8)	513 (44.2)
Education, %				
0-8 years	33 (0.5)	10 (0.3)	9 (0.4)	14 (1.2)
Some high school	3521 (48.5)	2213 (55.8)	924 (43.4)	384 (33.1)
High school diploma/GED	1031 (14.2)	469 (11.8)	327 (15.4)	235 (20.2)
Some higher education	2507 (34.5)	1224 (30.9)	809 (38.0)	474 (40.8)
College degree or higher	165 (2.3)	51 (1.3)	60 (2.8)	54 (4.7)
Family income, % [c]				
< $10,000	635 (9.3)	245 (6.6)	205 (10.2)	185 (17.0)
$10,000 - $19,999	1500 (22.0)	721 (19.4)	458 (22.8)	321 (29.6)
$20,000 - $34,999	1455 (21.4)	767 (20.6)	462 (23.0)	226 (20.8)
$35,000 - $49,999	1462 (21.5)	876 (23.5)	419 (20.9)	167 (15.4)
$50,000 - $74,999	1601 (23.5)	1075 (28.9)	410 (20.4)	116 (10.7)
$75,000 +	161 (2.4)	39 (1.0)	51 (2.5)	71 (6.5)
Charlson Index mean (sd)	0.32 (0.61)	0.22 (0.49)	0.34 (0.61)	0.61 (0.82)
History of rheumatoid arthritis, %	311 (4.3)	123 (3.1)	107 (5.0)	81 (7.1)
History of congestive heart failure %	52 (0.7)	9 (0.2)	17 (0.8)	26 (2.3)

© 2020 Cespedes Feliciano EM et al. *JAMA Network Open*.

Emphysema, %	250 (3.5)	77 (1.9)	72 (3.4)	101 (8.7)
COPD, %	732 (10.1)	299 (7.5)	221 (10.4)	212 (18.3)
Liver disease ever, %	164 (2.3)	86 (2.2)	46 (2.2)	32 (2.8)

[a] Characteristics measured at the 3-year visit following enrollment into the Women's Health Initiative

[b] Fatigue measured on Research ANd Development Corporation Short Form (RAND SF) -36 vitality and physical functioning scales

[c] Household size not accounted for

© 2020 Cespedes Feliciano EM et al. *JAMA Network Open*.

eFigure. Timelines for Frailty Data Collection beginning at Women's Health Initiative Enrollment Onwards and for Analysis of Time to Death following Cancer Diagnosis

A. Timeline of data collection in the Women's Health Initiative for frailty score and updates to specific score components; eligibility criteria for this analysis included at least two frailty measurements (enrollment and 3-year visit) prior to cancer diagnosis

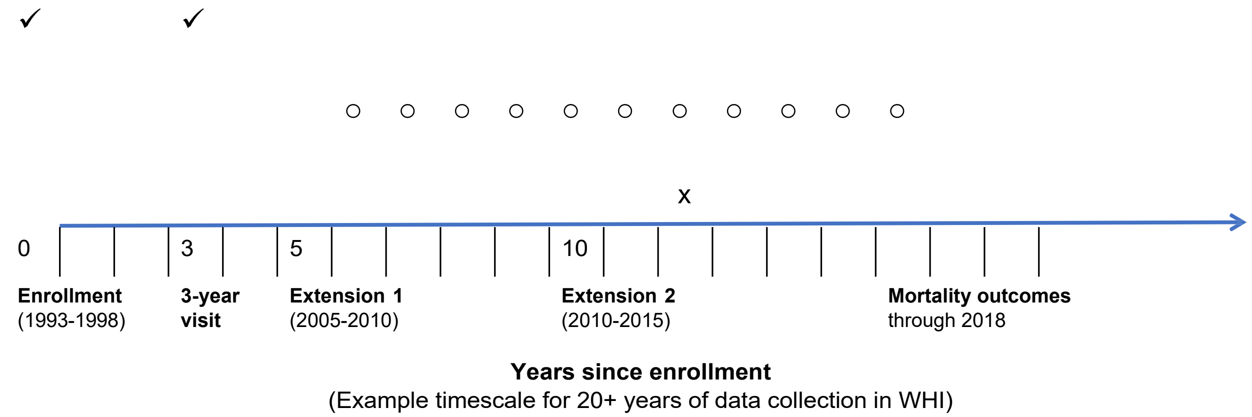

B. Sample analytic timeline for three individuals with frailty measured at variable times in relation to cancer diagnosis who are then followed forward from cancer diagnosis for mortality outcomes

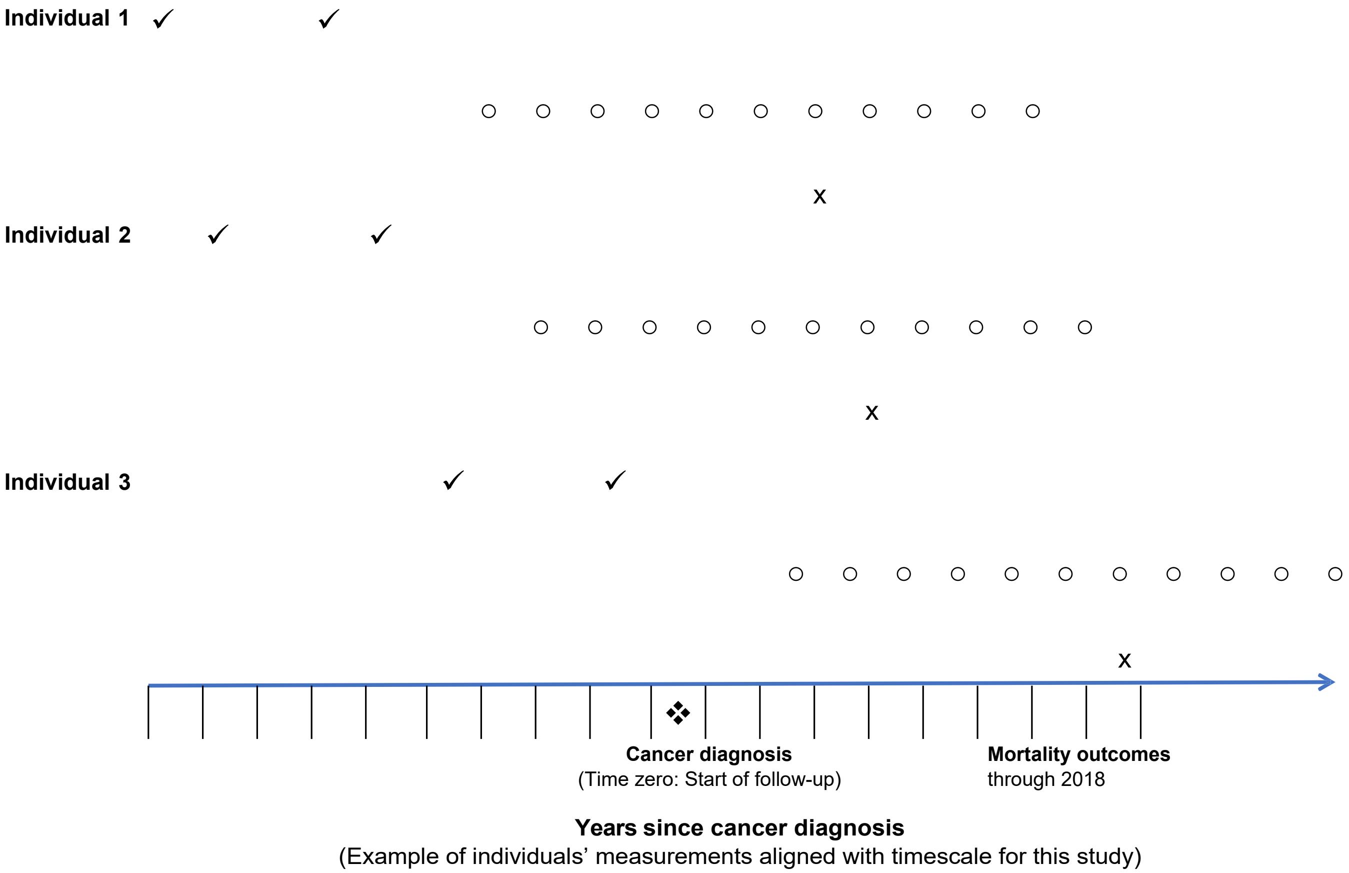

✓ = Full frailty score updated at enrollment and 3-year visit (cancer cases occurring prior to this are excluded)

○ = Physical functioning components updated annually during extension studies

x = Energy and fatigue component updated once during extension studies in 2012

❖ = Cancer diagnosis (time zero for this analysis)

© 2020 Cespedes Feliciano EM et al. *JAMA Network Open*.
